# Physiological and Transcriptomic Responses to Cold Stress in Taiwan Loach (*Paramisgurnus dabryanus* ssp. Taiwan)

**DOI:** 10.3390/antiox15081022

**Published:** 2026-08-17

**Authors:** Wei Zhou, Jiale Chen, Yacheng Hu, Dezhi Li, Tengfei Yu, Huaishun Shen

**Affiliations:** 1 Wuxi Fisheries College, Nanjing Agricultural University, Wuxi 214081, China; 2022113011@stu.njau.edu.cn (W.Z.); 2023113013@stu.njau.edu.cn (J.C.); 2025813057@stu.njau.edu.cn (T.Y.); 2 Key Laboratory of Integrated Rice-Fish Farming Ecology, Ministry of Agriculture and Rural Affairs, Freshwater Fisheries Research Center, Chinese Academy of Fishery Sciences, Wuxi 214081, China; 3 Chinese Sturgeon Research Institute, China Three Gorges Corporation, Yichang 443100, China; hu_yacheng@ctg.com.cn; 4 College of Fisheries and Life Sciences, Dalian Ocean University, Dalian 116023, China; lidezhifafu@163.com

**Keywords:** cold stress, Taiwan loach, RNA-Seq, oxidative stress, gene expression

## Abstract

The Taiwan loach (*Paramisgurnus dabryanus* ssp. Taiwan) is a popular cultured fish in southern China due to its rich nutritional content and rapid growth. Oxidative stress and homeostatic imbalance induced by low temperature are among the key factors restricting its large-scale aquaculture. Therefore, it is of great significance to investigate the oxidative stress damage and the adaptive mechanisms employed by this species in response to low temperature. In this study, the water temperature was lowered from 24 °C to 8 °C at a rate of 2 °C/h. Afterwards, the temperature was held at 8 °C for two durations: 12 h and 48 h. Antioxidant indices indicated that the Taiwan loach suffered from oxidative stress damage at the 12 h stage, but the antioxidant enzyme defense system was not fully activated. As the cold stress extended to 48 h, the activities of total superoxide dismutase (T-SOD), glutathione peroxidase (GSH-Px) and catalase (CAT) increased significantly (*p* < 0.01). Histological observations revealed that the livers exhibited cellular vacuolation, sinusoid congestion and karyolysis under cold stress. Transcriptomic data revealed that the Taiwan loach underwent adaptive alterations in response to low temperature through diverse pathways. We speculate that, at low temperature, the Taiwan loach may, on the one hand, regulate lipid metabolism to maintain cell membrane fluidity and meet energy demands, and, on the other hand, reprogram protein synthesis and processing to avert the overaccumulation of misfolded proteins. In addition, it also adopts a strategy of lysine and polyamine accumulation. This study provides a novel theoretical basis for breeding cold-tolerant varieties of Taiwan loach and optimizing overwintering aquaculture management.

## 1. Introduction

As a key abiotic factor in aquaculture, water temperature modulates multiple life activities of fish. Drastic shifts in temperature may even lead to the death of aquatic organisms [1]. Owing to climate change, temperature fluctuations have become increasingly difficult to predict. Southern China often suffers from abrupt temperature fluctuations during the autumn–winter transition. These fluctuations can lead to physiological damage in aquatic animals and the sudden onset of diseases, thereby causing significant economic losses to aquaculture.

Low temperatures are known to frequently induce oxidative stress in aquatic animals [2]. Through long-term evolution, aquatic species have developed a sophisticated antioxidant defense system to counteract oxidative damage. Among its components, superoxide dismutase (SOD), catalase (CAT), and glutathione peroxidase (GSH-Px) can effectively scavenge excess reactive oxygen species (ROS) through enzymatic reactions, while malondialdehyde (MDA), as a downstream product of lipid peroxidation, is commonly used as an indicator to assess the level of oxidative stress experienced by the organism [3]. Chen et al. observed that exposure to 10 °C significantly elevated CAT activity and MDA content but reduced T-SOD and GSH-Px activities in freshwater drum (*Aplodinotus grunniens*), indicating that the antioxidant system may provide partial protection against oxidative damage caused by low temperature [4]. After 7 and 14 days of low-temperature exposure in fourfinger threadfin (*Eleutheronema tetradactylum*), the activities of all aforementioned antioxidant enzymes all increased first and then decreased. Long-term cold stress eventually exhausted the compensatory capacity of its antioxidant enzyme system [5]. Overall, the responses of the antioxidant enzyme system to low temperature vary among different fish species.

As a robust research strategy, transcriptomic analysis relies on the continuous development and iteration of sequencing technologies. Currently, second-generation sequencing (NGS) serves as the prevailing tool for transcriptomic profiling, which facilitates robust excavation of transcriptomic signatures in organisms without available reference genomic resources [6]. Lipid metabolism represents a key research subject in transcriptomic studies of cold stress in fish. Yang et al. investigated the cold adaptation of burbot (*Lota lota*) and identified multiple DEGs related to fatty acid metabolism. They found that the gene encoding fatty acid desaturase (FAD) was significantly upregulated, indicating that unsaturated fatty acid synthesis contributes to cold tolerance in this species [7]. A multi-tissue transcriptomic analysis of tiger barb (*Puntius tetrazona*) under cold stress also revealed extensive enrichment of fatty acid-related pathways [8]. In addition, protein synthesis and processing are also crucial for cold adaptation in fish. In yellowtail kingfish (*Seriola aureovittata*), analysis under cold stress showed strong enrichment of the protein processing in endoplasmic reticulum pathway, and significant downregulation of calreticulin (*CALR*) was proposed to reduce excessive accumulation of misfolded proteins [9]. He et al. found that hub genes associated with ribosome and spliceosome pathways were significantly upregulated in *Acrossocheilus wenchowensis* under cold stress [10]. These studies have provided abundant data support for deciphering the molecular mechanisms underlying cold acclimation in fish.

The total aquaculture output of loach in China reached 278,332 tons in 2024, ranking 17th among all freshwater farmed fish species [11]. Taiwan loach (*Paramisgurnus dabryanus* ssp. Taiwan), a local population of large-scale loach (*Paramisgurnus dabryanus*), displays better growth traits than the true loach (*Misgurnus anguillicaudatus*) [12]. In addition, the Taiwan loach is valued for its high protein, abundant essential amino acids, and low lipid content [13]. Therefore, in some provinces of China, like Zhejiang, Guangdong, and Jiangxi, it is widely cultivated [14]. However, Taiwan loach exhibits weaker cold tolerance than the true loach [15]. Accordingly, exploring its adaptive mechanisms under cold stress is of great significance for the popularization of Taiwan loach aquaculture in cold northern regions of China. Upon measuring antioxidant enzyme activities in Taiwan loach exposed to cold stress, Tao et al. observed severe physiological damage along with signs of certain adaptive responses [16]. Fan et al. conducted a cold stress and recovery experiment on large-scale loach, measuring antioxidant and innate immune parameters. Their results showed that low temperature suppressed both capacities, but these parameters recovered as temperature increased [17]. To date, the hepatic transcriptomic responses of Taiwan loach to cold stress have not been investigated. Hence, this study aimed to elucidate the cold response mechanisms of Taiwan loach by integrating transcriptomic, histological and antioxidant analyses of the liver following 12 h and 48 h of cold exposure at 8 °C. Our findings provide a theoretical basis for breeding cold-tolerant strains and optimizing overwintering aquaculture management.

## 2. Materials and Methods

### 2.1. Source of Samples and Experimental Design

A total of 250 adult Taiwan loaches were collected from Huzhou, Zhejiang Province, China and cultivated at 24 ± 0.2 °C for two weeks before the experiment. During the acclimation period, natural light conditions were maintained, and commercial compound feed was supplied three times daily. Feeding ceased one day before the trial commenced. A total of 180 healthy and comparably sized loaches were selected for the study and randomly assigned to six tanks. The low-temperature treatment group was subjected to a gradual decrease in temperature from 24 °C to 8° C at a rate of 2 °C per hour. The temperature was then maintained at 8 °C for 12 h (LT1) and 48 h (LT2). Taiwan loaches were sampled from control and cold-exposed groups at the LT1 and LT2 time points, respectively. The sample size design of this study follows the common sampling protocols reported in previous fish cold stress studies [18,19]. During sampling, individuals from the control group and low-temperature treatment groups were collected alternately. Following the administration of MS222 to anaesthetize the Taiwan loach, the liver was rapidly extracted for analysis. Partial liver tissues were fixed in 4% paraformaldehyde solution for HE staining. The remaining samples were immediately frozen at −80 °C to detect antioxidant enzyme activities and perform transcriptome sequencing. All animal experiments and sample detections described in this study were completed at the Freshwater Fisheries Research Center, Wuxi.

### 2.2. Antioxidative Parameters Analysis

Six Taiwan loach individuals were randomly selected from each group, and their liver tissues were harvested for subsequent biochemical assays. The activities of key antioxidant enzymes and MDA content were measured using kits from Nanjing Jiancheng Bioengineering Co., Ltd. (Nanjing, China). Specifically, T-SOD activity was quantified using the hydroxylamine method (Cat No. A001-1-2); MDA content was determined via the TBA assay (Cat No. A003-1-2); CAT activity was measured with the visible light method (Cat No. A007-2); and GSH-Px activity was assessed using a microplate-based approach (Cat No. A006-2-1). The absorbance values of biochemical indicators were measured using a Multiskan GO microplate spectrophotometer (Thermo Scientific, Waltham, MA, USA).

### 2.3. Histological Staining Observations

Three fish were assigned to each group as biological replicates, with two tissue sections prepared per individual fish. Liver tissue was processed through a gradient of ethanol and xylene, then embedded in paraffin. The resulting paraffin samples were sectioned into 4 µm sections and mounted onto glass slides. After sectioning, the slides were deparaffinized in xylene, then rehydrated in graded ethanol. HE staining was performed, and the sections were visualized under a Leica DM2000 upright optical microscope (Leica Microsystems, Wetzlar, Germany). Representative images were chosen from clear, artifact-free sections with intact tissue architecture, whose pathological features were consistent with the dominant lesions observed in most sections of the same group.

### 2.4. Analysis of Transcriptome and DEGs

Three biological replicates were established per group, and each replicate contained pooled liver tissues collected from three Taiwan loaches. Total RNA was isolated with TRIzol (Invitrogen, Carlsbad, CA, USA), and integrity was assessed with an Agilent 2100 Bioanalyzer (Agilent Technologies, Santa Clara, CA, USA).

Poly(A) mRNA was enriched by poly-T magnetic beads, then fragmented in Fragmentation Buffer with divalent cations to construct cDNA libraries. Libraries were quantified with a Qubit 2.0 fluorometer (Thermo Fisher Scientific, Waltham, MA, USA) and checked for insert size on an Agilent 2100 Bioanalyzer. Illumina NovaSeq 6000 platform was used for sequencing the qualified libraries (Novogene Co., Ltd., Beijing, China). To ensure analytical accuracy, raw reads were filtered to obtain high-quality clean data. Transcripts were assembled using Trinity [20] and subsequently subjected to hierarchical clustering with the Corset [21]. In order to obtain comprehensive gene functions information, all transcripts were compared with following seven databases: Non-Redundant protein database (Nr), Nucleotide sequence database (Nt), Pfam protein family database (Pfam), Eukaryotic Orthologous Groups database (KOG), Swiss-Prot protein database (Swiss-Prot), Kyoto Encyclopedia of Genes and Genomes (KEGG), and Gene Ontology (GO).

### 2.5. qRT-PCR Validation of RNA-Seq Data

For quantitative real-time polymerase chain reaction (qRT-PCR) validation of eight selected DEGs, total RNA was isolated with TRIzol reagent, and cDNA was prepared using the PrimeScript™ RT reagent Kit (TaKaRa, Kusatsu, Japan). *RPS27* was employed as the internal reference gene for qRT-PCR. The CFX96 system (Bio-Rad, Hercules, CA, USA) was used for qRT-PCR, and relative gene expression was calculated by the 2^−ΔΔCT^ method. The amplification program was 95 °C for 3 min, followed by 40 cycles of 95 °C for 10 s and 60 °C for 30 s. Primers were designed using Primer 5.0 (Table S1) and obtained from Shanghai Generay Biotech Co., Ltd. (Shanghai, China).

### 2.6. Statistical Analysis

Statistical analyses of the antioxidative parameters were performed using SPSS 26.0. Homogeneity of variances was evaluated using Levene’s test, which confirmed equal variances across all comparisons. The Shapiro–Wilk test was used to assess the normality. For datasets meeting the normality assumption, comparisons between groups were conducted using the Independent Samples *t*-test. For datasets that deviated from normality, the non-parametric Mann–Whitney U test was applied. A significance level was defined as 0.01 < *p* < 0.05, and an extremely significant level was defined as *p* < 0.01. DEGs were identified using DESeq2 (version 1.20.0) with the criteria of Q-value < 0.05 and |log_2_FoldChange| > 1. For each detection assay, all randomly selected individuals were incorporated into the final analysis, and no data points were excluded on the grounds of outliers.

## 3. Results

### 3.1. Impact of Temperature Stress on Antioxidant-Related Indexes

**Figure 1:**
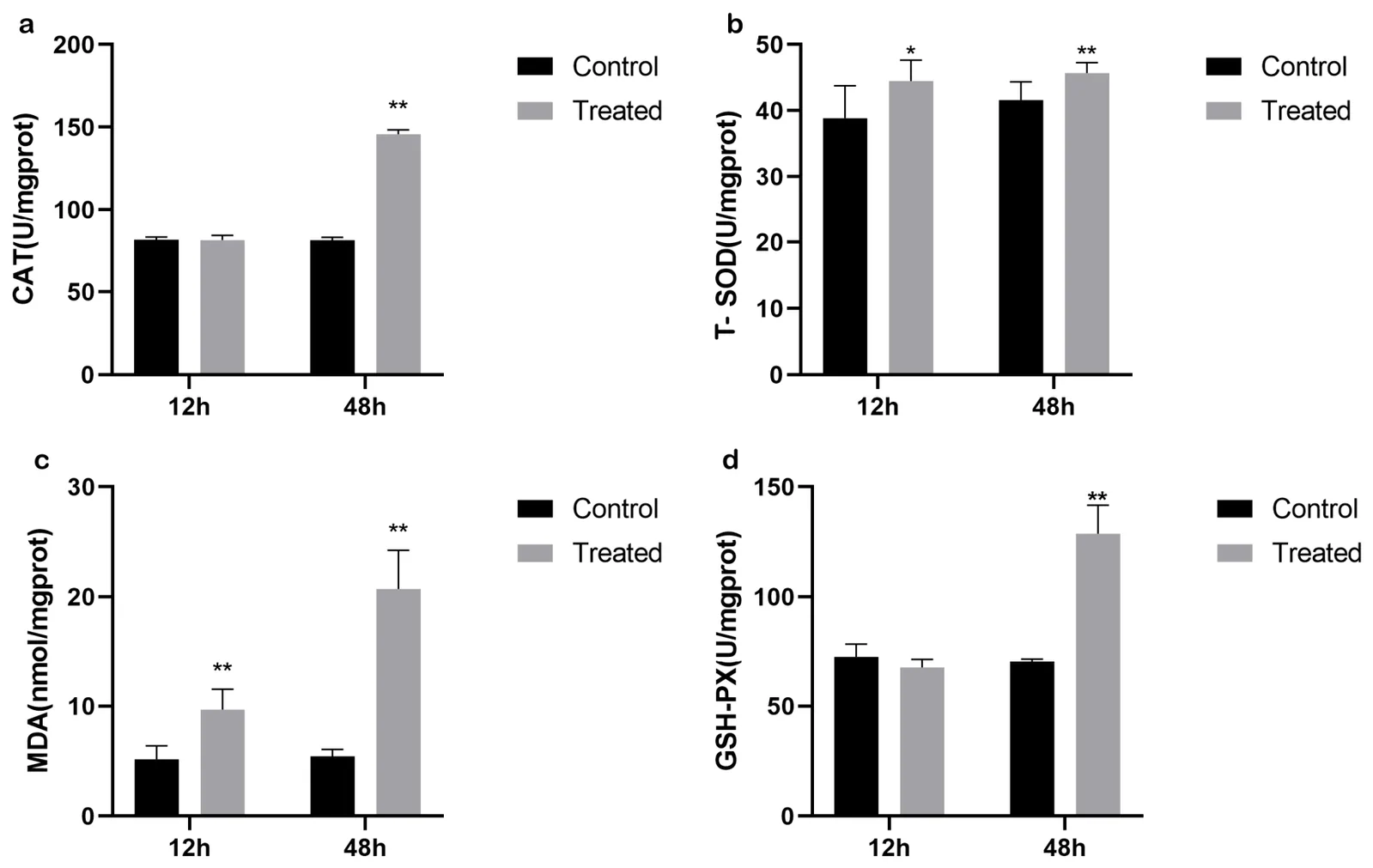
Effects of cold stress on antioxidant parameters in Taiwan loach. (**a**), CAT; (**b**), T-SOD; (**c**), MDA; (**d**), GSH-Px. Significant differences from the control are marked with * (*p* < 0.05) and ** (*p* < 0.01).

After 12 h of cold stress at 8 °C, CAT and GSH-Px activities in Taiwan loaches were similar to the control group (*p* > 0.05). Meanwhile, T-SOD activity increased significantly (*p* < 0.05), and MDA content was also significantly higher (*p* < 0.01). As the cold stress time extended to 48 h, the antioxidant response became more pronounced. The activities of all three major antioxidant enzymes were significantly increased (*p* < 0.01), suggesting a coordinated upregulation of the enzymatic defense system to combat oxidative stress. Consistently, MDA content also remained significantly increased (*p* < 0.01), reflecting sustained lipid peroxidation under continuous low-temperature conditions. The detailed results are shown in Figure 1 and Table S2.

### 3.2. Morphological Observations

**Figure 2:**
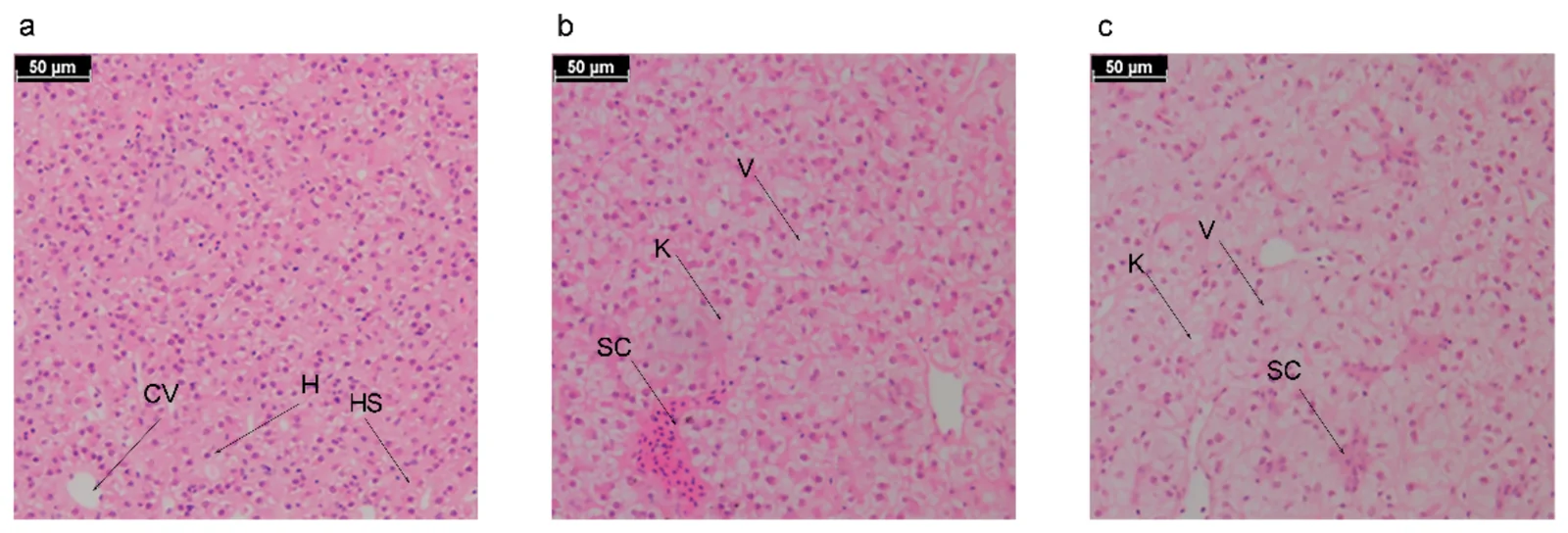
HE staining of liver tissue sections. (**a**), control group; (**b**), LT1 group; (**c**), LT2 group. H, hepatocyte; CV, central vein; HS, hepatic sinusoid; V, vacuolation; K, karyolysis; SC, hepatic sinusoid congestion. Scale bar = 50 μm.

Liver tissues from the control group displayed typical normal histological characteristics. Hepatocytes were orderly arranged with dense cell distribution, showing regular cell morphology. Additionally, the central venous structure was well-developed and free from pathological abnormalities, which represented the healthy state of hepatic tissue (Figure 2a). Histological analysis using HE staining revealed that, following 12 h of cold stress at 8 °C, the low-temperature treatment group exhibited cellular vacuolation, hepatic sinusoid congestion, and karyolysis compared to the control group (Figure 2b). These histopathological changes persisted after 48 h of treatment (Figure 2c). The above morphological changes were consistently observed in all three biological replicates at both 12 h and 48 h of cold exposure.

### 3.3. RNA-Seq Data Quality and DEGs in Hepatic Response to Low-Temperature Stress

**Figure 3:**
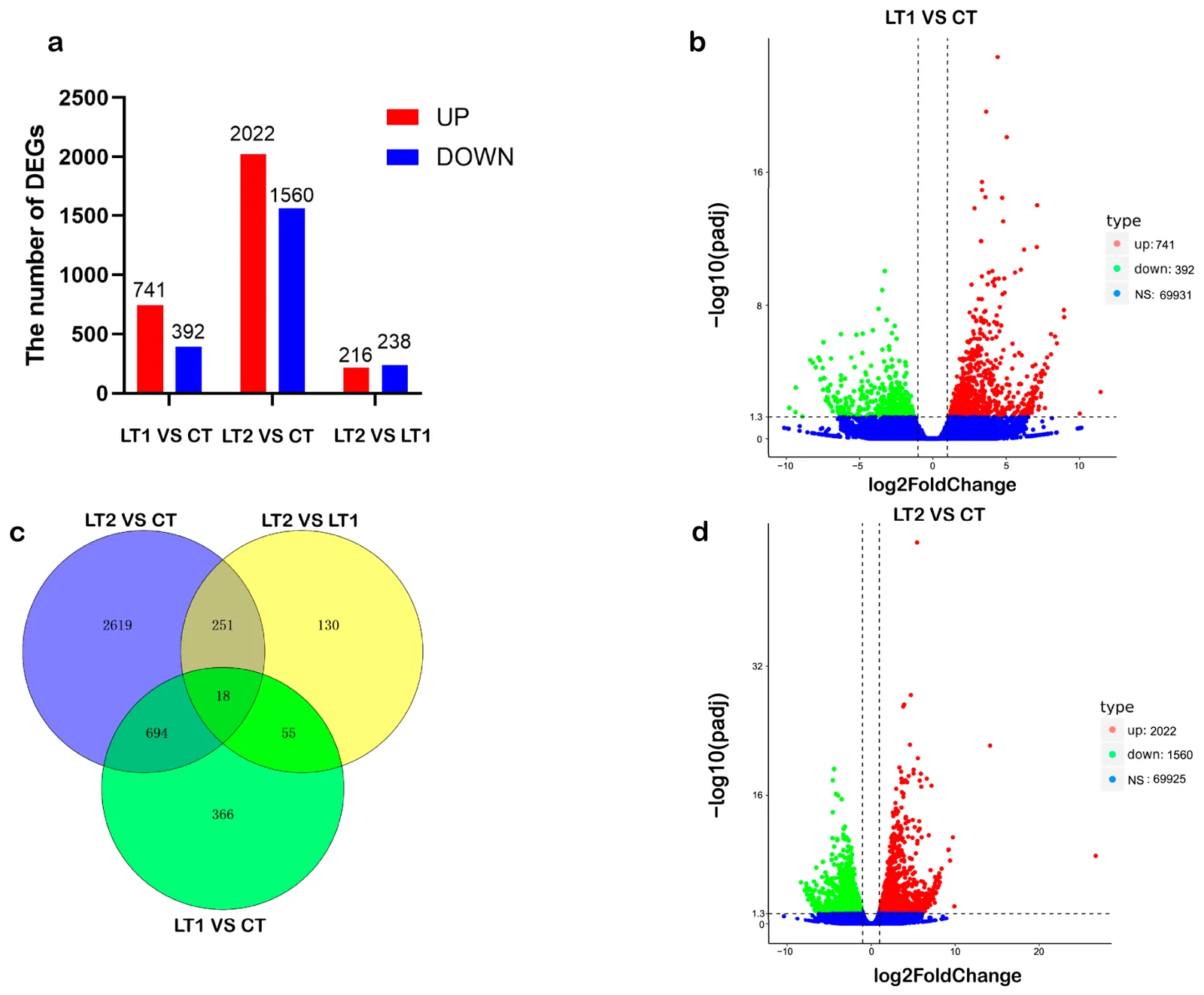
Summary of DEGs under cold stress. (**a**), number of upregulated (red bars) and downregulated (blue bars) DEGs; (**b**), volcano plot of DEGs in the LT1 vs. CT comparison; (**c**), Venn diagram showing the overlap of DEGs; (**d**), volcano plot of DEGs in the LT2 vs. CT comparison. In subfigures (**b**,**d**), red dots represent significantly upregulated genes, green dots represent significantly downregulated genes, and blue dots represent non-significant (NS) genes.

In total, 187,651,938 clean reads were generated from nine liver transcriptomes. All cDNA libraries had Q30 values above 94%, with GC content ranging from 45.29% to 46.92% (Table 1). Transcriptomic analysis revealed 1133 DEGs in the LT1 group, comprising 741 upregulated and 392 downregulated genes (Figure 3a,b). In the LT2 group, 3582 DEGs were detected, with 2022 upregulated and 1560 downregulated (Figure 3a,d). In addition, a comparative analysis between LT1 and LT2 revealed 454 DEGs, including 216 upregulated and 238 downregulated genes (Figure 3a). Among all pairwise comparisons, 18 DEGs were commonly identified across all three groups (Figure 3c).

### 3.4. GO and KEGG Enrichments Analysis of DEGs

**Figure 4:**
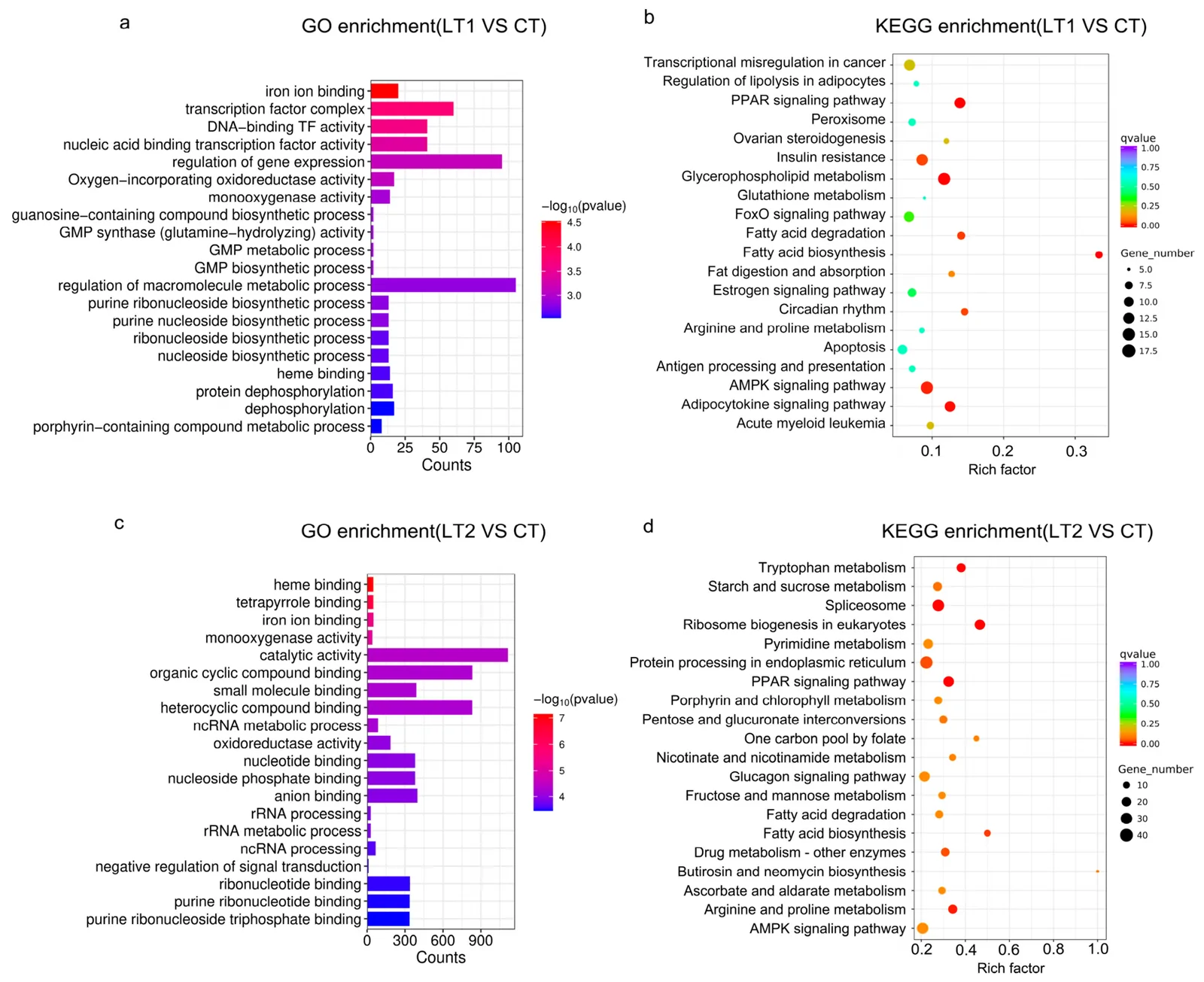
GO and KEGG enrichment analyses of DEGs under cold stress. (**a**), GO enrichment of DEGs in LT1 vs. CT; (**b**), KEGG enrichment of DEGs in LT1 vs. CT; (**c**), GO enrichment of DEGs in LT2 vs. CT; (**d**), KEGG enrichment of DEGs in LT2 vs. CT.

The top 20 significantly enriched GO terms in the LT1 group contained 7 molecular function (MF), 1 cellular component (CC), and 12 biological process (BP) entries. The top enriched GO terms were primarily associated with transcriptional regulation, purine/GMP nucleotide metabolism, heme/iron-dependent redox activities and cofactor binding, and protein dephosphorylation (Figure 4a). In the LT2 group, the top 20 significantly enriched GO terms included 15 MF and 5 BP, and they were primarily associated with catalytic/redox activities, heterocyclic/small molecule binding, RNA metabolic processes, and nucleotide/ribonucleotide binding (Figure 4c).

According to KEGG pathway enrichment, DEGs in LT1 were significantly associated with fatty acid biosynthesis, PPAR signaling pathway, glycerophospholipid metabolism, adipocytokine signaling pathway, AMPK signaling pathway, fatty acid degradation, circadian rhythm, and insulin resistance (Figure 4b, Table S3). Meanwhile, DEGs in LT2 were enriched in ribosome biogenesis in eukaryotes, PPAR signaling pathway, spliceosome, tryptophan metabolism, arginine and proline metabolism, fatty acid biosynthesis, protein processing in endoplasmic reticulum, and drug metabolism—other enzymes (Figure 4d, Table S3). Eukaryotic ribosome biogenesis pathway showed the most significant enrichment, exhibiting the lowest adjusted *p* value (6.70 × 10^−6^) and involving 34 DEGs. This indicates a strong activation in response to cold stress (Table S3).

### 3.5. Validation of RNA-Seq Results by qRT-PCR

**Figure 5:**
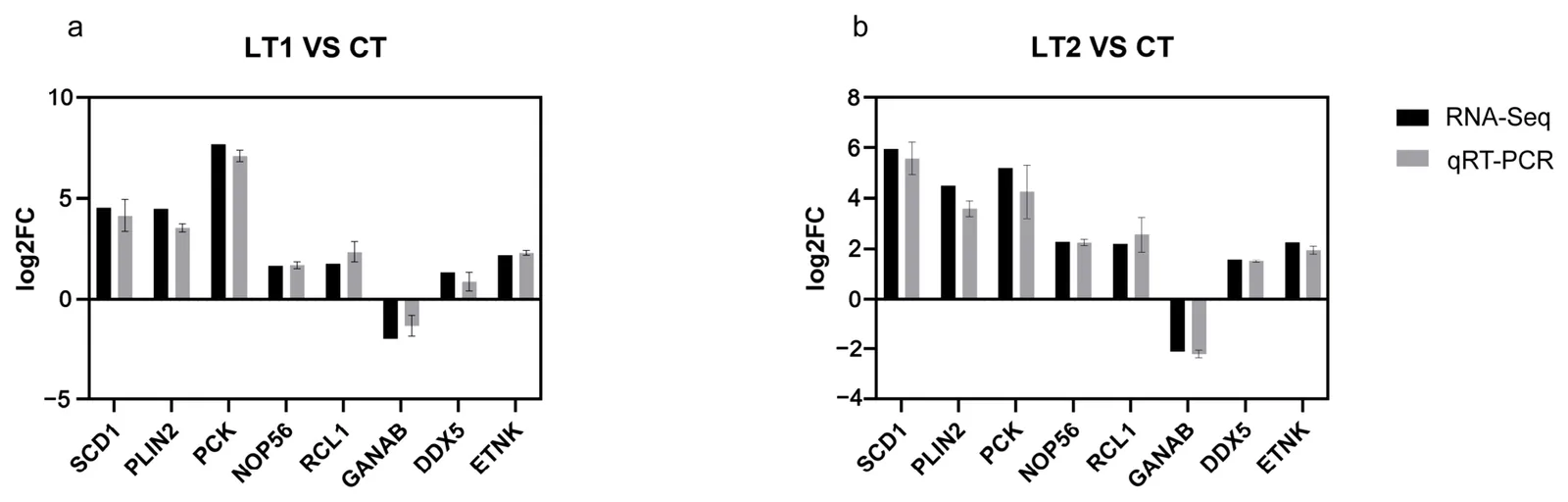
Validation of RNA-Seq results by qRT-PCR. (**a**), LT1 vs. CT; (**b**), LT2 vs. CT. The x-axis represents genes and the y-axis represents log_2_ fold change.

To verify the reliability of RNA-Seq results, eight candidate DEGs were selected for qRT-PCR quantification in Taiwan loach. The consistent expression trends obtained from qRT-PCR and RNA-Seq confirmed the validity of the transcriptomic data (Figure 5).

## 4. Discussion

Cold stress exerts multifaceted effects on fish, often accompanied by physiological stress responses, metabolic changes, and alterations in immune function [22]. Understanding the molecular and physiological responses to cold stress is essential for developing resilient aquaculture species and sustainable management practices, particularly in regions experiencing seasonal or climate-induced temperature variability [1]. The liver, a key organ for metabolism, detoxification, and immune regulation, is an ideal target for studying low-temperature stress responses in aquatic organisms [23]. Therefore, to investigate the adaptive mechanisms of Taiwan loach to cold stress, the loach was exposed to 8 °C, and liver tissue was collected for integrated histological, antioxidant, and transcriptomic analyses.

To alleviate further damage caused by oxidative stress, organisms activate an antioxidant defense system [24]. As a ubiquitous enzyme in aquatic organisms, T-SOD mediates the transformation of superoxide anions into hydrogen peroxide. CAT and GSH-Px further transform hydrogen peroxide into water and oxygen, thereby protecting cellular function and maintaining organismal stability [25]. At the 12 h sampling point of cold stress in Taiwan loaches, only T-SOD activity increased significantly (*p* < 0.05). This indicates that the antioxidant enzyme system was not fully activated to counteract oxidative stress during the early stage of cold exposure. By 48 h, all three antioxidant indices significantly increased (*p* < 0.01), suggesting enhanced antioxidant capacity. In the study by Tao et al. on Taiwan loach, these three parameters initially decreased and then increased with the duration of cold stress, which may be attributed to differences in experimental design and conditions [16]. MDA, a product of ROS-induced lipid peroxidation, is an indirect biomarker of oxidative stress damage [26]. In the Taiwan loach experiment, liver MDA levels significantly increased at both 12 and 48 h (*p* < 0.01), indicating sustained oxidative stress damage. Overall, despite the activation of antioxidant enzymes, oxidative damage in the liver was not effectively reversed. In addition, the detection of more physiological indicators such as ROS and protein carbonyl would help comprehensively characterize the oxidative stress adaptation of Taiwan loach under cold stress. Hepatic damage induced by low temperature has been widely documented in previous fish studies. For instance, massive loss of cell nuclei was observed in the liver of juvenile hybrid sturgeon under cold stress [3]. Relevant research also reported obvious nuclear swelling and disordered cell arrangement with severe hepatic lesions in cold-sensitive black seabream (*Acanthopagrus schlegelii*), while only mild hepatocellular damage was detected in cold-tolerant individuals [27]. HE staining revealed that liver tissue damage persisted as cold stress was prolonged. Severe vacuolation greatly reduces the functional volume of hepatocytes, which further impairs the metabolic, synthetic and detoxification capacities of the liver. Extensive karyolysis indicates widespread necrotic death of hepatic parenchymal cells [28]. Overall, our results demonstrate that cold stress triggers irreversible structural destruction in hepatocytes, highlighting the critical need for strict water temperature management and thorough pre-winter preparation in Taiwan loach aquaculture. In addition, quantitative histomorphometry would deliver more intuitive intergroup comparative evidence, even though our qualitative histological observation has reflected several abnormal structural changes.

The PPAR signaling pathway, a key regulator of lipid metabolism, has been widely implicated in cold stress responses in various fish species, such as red-tail catfish (*Hemibagrus wyckioides*) [29], freshwater drum (*Aplodinotus grunniens*) [30], shortbarbel schizothoracin (*Schizothorax wangchiachii*) [31] and yellow drum (*Nibea albiflora*) [32]. Stearoyl-CoA desaturase 1 (SCD1) is the rate-limiting enzyme for monounsaturated fatty acid synthesis and catalyzes the desaturation of saturated fatty acids [33]. Carnitine palmitoyltransferase 1 (CPT1) modulates fatty acid β-oxidation, while Phosphoenolpyruvate carboxykinase (PCK) acts as a key gluconeogenic enzyme that drives glucose synthesis from non-carbohydrate precursors [34]. In the LT1 group, the genes encoding these three enzymes showed significant transcriptional upregulation. We hypothesize that the increased transcript abundance of these genes may serve as a potential molecular preparation for membrane fluidity maintenance and enhanced lipid catabolism under acute cold stress. In a study on cobia (*Rachycentron canadum*) under cold stress, Cai et al. observed elevated expression of *SCD1* and *CPT1*, a pattern similar to what we detected in this work [35]. Zhang et al. also found that *SCD1* expression was significantly upregulated in silver pomfret (*Pampus argenteus*) under low temperature, and they also observed significant upregulation of long-chain acyl-CoA synthetase (*ACSL*), suggesting that fish increase fatty acid utilization under cold stress [36]. In LT2, sustained cold stress led to the downregulation of peroxisome proliferator-activated receptor alpha (*PPARα*) and retinoid X receptor (*RXR*), along with reduced expression of their target gene *CPT2* and bile acid synthesis-related genes. Concurrently, genes associated with lipid storage and gluconeogenesis, including *SCD1*, perilipin 1 (*PLIN1*), *PCK*, and angiopoietin-like 4 (*ANGPTL4*), were upregulated. This expression pattern may imply a metabolic shift from fatty acid oxidation toward lipid conservation during prolonged cold exposure. We hypothesize that this transcriptional shift aims to moderately suppress fatty acid oxidation to reduce sustained mitochondrial ROS production, so as to alleviate cold-induced oxidative damage and retain the body’s lipid energy reserves. However, MDA levels in the 48 h cold treatment group were still significantly higher than those of the control group (*p* < 0.01), demonstrating that lipid peroxidation damage was not effectively alleviated. Massive accumulated lipid peroxides damage the integrity of the Endoplasmic reticulum (ER) membrane and disturb ER redox homeostasis, which may serve as one of the key triggers for protein quality control-centered transcriptional reprogramming in the LT2 group [37]. Wang et al. also observed similar phenomena in a cold stress experiment on striped catfish (*Pangasianodon hypophthalmus*) [38]. Overall, PPAR pathway plays a critical role in the response of Taiwan loach to low temperature.

Under cold stress, biological cell membranes tend to shift from a liquid-crystalline state to a gel-like non-fluid state. Organisms counteract this deleterious transition through adaptive remodeling of membrane lipid composition, involving alterations in fatty acyl chain unsaturation, chain length, and phospholipid head-group composition, processes in which glycerophospholipid metabolism plays a central role [33]. Zhao et al. conducted a multi-omics study and found that in cobia (*Rachycentron canadum*), under cold stress, key metabolites in glycerophospholipid metabolism, including phosphocholine, CDP-choline, and glycerophosphocholine (GPC), were upregulated [39]. We found that in the LT1 group, the genes involved in the synthesis of phosphatidylcholine (PC) and phosphatidylethanolamine (PE), including lysophosphatidylcholine acyltransferase 3 (*LPCAT3*), lysophosphatidylcholine acyltransferase 4 (*LPCAT4*), phosphatidylserine decarboxylase (*PISD*), ethanolaminephosphotransferase 1 (*EPT1*), ethanolamine kinase (*ETNK*) and choline kinase (*CHK*), were all significantly upregulated. Notably, LPCAT3 participates in the Lands’ cycle and preferentially incorporates polyunsaturated fatty acids (PUFAs) into lysophosphatidylcholine (LPC), thereby generating PUFA-enriched PC [40]. In contrast, LPCAT4 selectively transfers monounsaturated fatty acids (MUFAs) to both LPC and lysophosphatidylethanolamine (LPE), driving the formation of MUFA-rich PC and PE molecular species [41]. Additionally, PE, due to its conical molecular structure, contributes to maintaining membrane curvature and fluidity [42]. Overall, these transcriptional alterations may increase the overall unsaturation of membrane fatty acyl chains, thereby counteracting the cold-induced phase transition from the liquid-crystalline to the gel state. In the LT2 group, genes involved in PE synthesis, including *PISD*, *EPT1*, CDP-diacylglycerol synthase 1 (*CDS1*), phosphate cytidylyltransferase 2 (*PCYT2*) and glycerophosphodiester phosphodiesterase domain containing 1 (*GPCPD1*), were upregulated. Phosphatidylethanolamine N-methyltransferase (*PEMT*) and ethanolamine phosphate phospholyase (*ETNPPL*), which are involved in PE-to-PC conversion and ethanolamine phosphate degradation, respectively, were downregulated. These transcriptional changes are consistent with a strategy to favor PE accumulation and limit precursor loss, potentially enhancing membrane fluidity under prolonged cold stress.

Small nuclear ribonucleoproteins (snRNPs) are core components of the spliceosome [43]. They recognize splice sites on pre-mRNA and participate in the splicing reaction [44]. Yu et al. reported that genes encoding snRNPs were significantly upregulated in giant river prawn (*Macrobrachium rosenbergii*) under cold stress. A similar transcriptional pattern was observed in our study [45]. In the LT2 group, we observed widespread upregulation of genes involved in spliceosome function. Among them, small nuclear ribonucleoprotein polypeptides D2 (*SNRPD2*), D3 (*SNRPD3*), and G (*SNRPG*) encode core subunits of snRNPs [46]. In addition, splicing factor 3A subunit 3 (*SF3A3*) and splicing factor 3B subunit 3 (*SF3B3*) serve to stabilize the interaction between U2 snRNA and pre-mRNA [47]. Low temperatures stabilize RNA secondary structures, which can mask splice sites and reduce spliceosome assembly efficiency [48,49]. The transcriptional activation of splicing machinery in the LT2 group likely constitutes a compensatory response to overcome such cold-induced splicing constraints. Consistent with this pattern, a study in the Przewalski’s naked carp (*Gymnocypris przewalskii*) reported that pooled DEGs obtained from eight sampled tissues exhibited significant enrichment in the spliceosome pathway under cold stress [50]. Similarly, in the Bombay duck (*Harpadon nehereus*), the spliceosome pathway was significantly enriched in both DEGs and differentially alternatively spliced genes (DASGs) across three low-temperature environments (13 °C, 15 °C, and 17 °C) [51]. Ribosomes serve as essential cellular sites for protein translation [52]. In the LT2 group, 34 genes involved in ribosome biogenesis in eukaryotes were significantly upregulated after cold stress. These genes span key processes including 90S pre-ribosome assembly, rRNA modification, and ribosomal subunit maturation. Such changes may indicate a compensatory response by cells to enhance protein synthesis capacity under low-temperature stress. Similarly, Long et al. observed significant upregulation of multiple genes related to the spliceosome and eukaryotic ribosome biogenesis pathways in zebrafish under cold stress. They proposed that this response may serve to counteract cold stress-induced suppression of protein folding [53].

ER stress can be induced by sharp environmental temperature changes, prompting organisms to activate the unfolded protein response (UPR) and endoplasmic reticulum-associated degradation (ERAD) to eliminate accumulated misfolded proteins [9,54]. In a report by Mininni et al., gilthead sea bream (*Sparus aurata*) under cold stress showed significant overexpression of genes including *ATF4*, *CHOP*, and *XBP1*, which may indicate activation of the UPR. However, hepatic ER stress remained unresolved under these conditions [55]. UPR is primarily mediated by inositol requiring enzyme 1 (IRE1), PKR-like ER kinase (PERK), and activating transcription factor 6 (ATF6) [56]. In the LT2 group, PERK-related transcripts showed preferential upregulation, whereas marker genes from the IRE1 and ATF6 branches exhibited decreased expression. We hypothesize that such transcriptional remodeling may render the UPR pathway a potential participant in cold stress adaptation. We also found significant upregulation of genes including ubiquitin-conjugating enzyme E2 J2 (*UBC6*), UBX domain-containing protein 1 (*UBX1*), valosin-containing protein (*VCP*), and ubiquitin-conjugating enzyme E2 G1 (*UBC7*). Among these, UBC6 initiates ubiquitination, and UBC7 catalyzes the elongation of polyubiquitin chains [57]. UBX1 and VCP participate in the membrane dislocation of ubiquitinated substrates [58]. This coordinated transcriptional response may reflect a potential priming of ERAD-related programs for misfolded protein clearance. We hypothesize that under sustained cold stress, Taiwan loach enhances protein synthesis by upregulating genes related to spliceosome function and ribosome biogenesis, which helps maintain essential cellular activities. At the same time, impaired protein folding capacity in the endoplasmic reticulum activates both the UPR and ERAD to prevent excessive accumulation of misfolded proteins. As a result, cold exposure may disrupt the dynamic balance between protein synthesis and degradation in this species. Of course, further verification is necessary to support this viewpoint. Sun et al. also reported similar findings in a low-temperature stress experiment on bombay duck (*Harpadon nehereus*) [51]. Although individual genes within these three pathways showed significant differential expression in the LT1 group, none of these pathways exhibited significant enrichment. Combined with antioxidant indices and histological observations, these results indicate that Taiwan loaches were already under stress at this stage, but had not yet activated protein synthesis-related adaptive strategies.

The arginine and proline metabolism pathway has been frequently reported in cold stress experiments across various fish species [59]. Yu et al. reported significant upregulation of ornithine decarboxylase (*ODC1*) and pyrroline-5-carboxylate reductase 1 (*PYCR1*) in largemouth bass (*Micropterus salmoides*) exposed to acute cold stress, and they speculated that the elevated transcription of these two key enzymes drives intracellular proline accumulation to mediate cold adaptation [60]. In our study, proline dehydrogenase (*PRODH*) and hydroxyproline dehydrogenase (*PRODH2*), which catalyze the first steps of proline and hydroxyproline catabolism, respectively, were significantly downregulated in the LT2 group [61]. This inhibition may reduce proline consumption and potentially promote its intracellular accumulation. Conversely, genes associated with polyamine synthesis, such as *ODC1*, spermine synthase (*SMS*), S-adenosylmethionine decarboxylase (*AMD1*), and spermine oxidase (*SMOX*), were markedly upregulated. In LT1, the pathway lacked significant enrichment, but early changes included upregulation of *ODC1* and downregulation of *P4HA*, hinting at nascent adjustments. Proline, a proteinogenic amino acid with distinctive conformational rigidity, participates in environmental stress responses across diverse organisms [62]. Polyamines are aliphatic compounds containing nitrogen, with the capacity to scavenge excess free radicals and preserve DNA integrity [63,64]. We speculate that such shifts in gene expression may facilitate the accumulation of proline and polyamines, thereby enhancing cold stress adaptation in Taiwan loach. Nevertheless, this inference requires supporting metabolomic data and further in-depth investigation for validation.

## 5. Conclusions

Hypothermia triggers oxidative stress and prominent transcriptomic changes in the liver of Taiwan loach. At the physiological level, MDA content and histological observations both indicated that cold exposure caused continuous liver damage in Taiwan loach. We also observed delayed activation of the antioxidant enzyme system. These findings highlight the limitations of antioxidant defenses in fully mitigating ROS-mediated damage under hypothermia. Transcriptomic analysis identified 1133 and 3582 DEGs at 12 h and 48 h, respectively, demonstrating an escalating molecular response. Early cold responses were characterized by widespread transcriptional upregulation of lipid metabolism pathways, especially the PPAR signaling pathway and glycerophospholipid metabolism. Such transcriptional changes may lay a molecular foundation for rapid membrane remodeling and energy mobilization at the physiological level. With prolonged cold stress, more complex transcriptional reprogramming was triggered, and differentially expressed genes are significantly enriched in pathways related to protein synthesis and quality control, alongside sustained modulation of lipid metabolism. Overall, Taiwan loach exhibits a certain degree of cold adaptation, yet persistent oxidative and hepatic damage suggests incomplete adaptation. These insights into phased molecular mechanisms provide a foundation for breeding cold-tolerant strains and improving aquaculture practices in thermally variable aquatic environments. Moreover, the application of emerging technologies such as spatial transcriptomics, alongside other multi-omics approaches and investigations of long-term cold adaptation, would further elucidate the spatiotemporal molecular regulatory networks underlying cold tolerance in Taiwan loach.

## Figures and Tables

**Table 1:** Summary of transcript sequencing results in Taiwan loach.

Sample	Raw Reads	Clean Reads	Q20/%	Q30/%	GC/%
CT-1	20,820,365	20,252,475	98.43	95.12	45.37
CT-2	21,605,205	20,498,304	98.38	94.99	46.07
CT-3	21,491,659	20,790,700	98.38	95.15	45.34
LT1-1	23,130,004	21,853,709	98.45	95.24	46.92
LT1-2	21,483,978	20,738,141	98.39	95.05	46.16
LT1-3	21,008,464	20,140,537	98.51	95.33	46.80
LT2-1	23,635,823	22,607,377	98.22	94.74	45.87
LT2-2	22,022,892	21,139,029	98.48	95.27	46.05
LT2-3	20,590,835	19,631,666	98.46	95.14	45.29

Note: CT represents the control group cultured at 24 °C; LT1 denotes the group subjected to 8 °C cold stress for 12 h; and LT2 represents the group subjected to 8 °C cold stress for 48 h. Each group included three independent biological replicates, marked as -1, -2 and -3 respectively.

## Data Availability

The original contributions presented in this study are included in the article/Supplementary Materials. Further inquiries can be directed to the corresponding author. The raw RNA-Seq reads have been deposited in the NCBI SRA under BioProject accession number PRJNA1467085.

## Supplementary Materials

The following supporting information can be downloaded at: https://www.mdpi.com/article/10.3390/antiox15081022/s1, Table S1: Primers used in qRT-PCR; Table S2: Hepatic antioxidant indicators in cold-stressed Taiwan loach; Table S3: KEGG pathway enrichment analysis of DEGs in Taiwan loach under cold stress.

## Abbreviations

The following abbreviations are used in this manuscript:

CAT	Catalase
DEGs	Differentially expressed genes
ER	Endoplasmic reticulum
ERAD	Endoplasmic reticulum-associated degradation
GO	Gene Ontology
GSH-Px	Glutathione peroxidase
KEGG	Kyoto Encyclopedia of Genes and Genomes
MDA	Malondialdehyde
Q20/Q30	The percentage of bases with Phred quality scores ≥ 20 or ≥30
T-SOD	Total superoxide dismutase
UPR	Unfolded Protein Response
