# Supplementary material for: Physiological and Transcriptomic Responses to Cold Stress in Taiwan Loach (Paramisgurnus dabryanus ssp. Taiwan)
Source: Antioxidants (Basel). 2026 Aug 17;15(8):1022. doi: 10.3390/antiox15081022 (PMC13509272; doi:10.3390/antiox15081022)
Supplement: Supplementary file 1 [file antioxidants-15-01022-s001.zip › Table S1.pdf]

Table S1. Primers used in qRT-PCR.

Gene	Primer	Oligonucleotide (5′-3′)
PLIN2	F	CGGTGGTCAGTGATGGAGTC
	R	TTCAGTGCTGTGTCCACTCC
SCD1	F	CCTCTCCGGATCTTTCTGGC
	R	GGTCCGCATCAGTCTCTGAG
PCK	F	GACTCAGGATCAGCGAGACG
	R	TGGCTTTGTCCCATTCCTCC
NOP56	F	CATCTCCCACGCTGGAAGTT
	R	CCTGAACAGGGCCTTCTCTG
RCL1	F	AGGTGCATTTCTCCTGTCCG
	R	GTTCCTCGGATCCGCTTGAT
GANAB	F	CGCACAAAACTGGAATGCCA
	R	CCTCTGTGACTGGGTGAACC
DDX5	F	CCGTGAACTGGCACAACAAG
	R	AACTCCCCTCTCCAGATCCC
ETNK	F	AGAGCTTCCAGGTTCTGCAC
	R	TTGAGTATCGAGAGCGCGTC
RPS27	F	AACATGCCTCTTGCGAAGGA
	R	GAGTTGGGGCTCTGTACGAG
