# Supplementary material for: Physiological and Transcriptomic Responses to Cold Stress in Taiwan Loach (Paramisgurnus dabryanus ssp. Taiwan)
Source: Antioxidants (Basel). 2026 Aug 17;15(8):1022. doi: 10.3390/antiox15081022 (PMC13509272; doi:10.3390/antiox15081022)
Supplement: Supplementary file 1 [file antioxidants-15-01022-s001.zip › Table S2.pdf]

Table S2. Hepatic antioxidant indicators in cold-stressed Taiwan loach.

Indicator	Time	Control<br>Mean ±SD	Treatment<br>Mean ±SD/ Median (IQR)	*p*
GSH-Px	12h	72.69±5.89	67.51±4.01	0.106
	48h	70.30±1.34	125.10 (4.56)	0.002
MDA	12h	5.17±1.21	9.69±1.86	0.001
	48h	5.43±0.63	20.71±3.54	<0.001
T-SOD	12h	38.87±4.90	44.49±3.17	0.044
	48h	41.62±2.76	46.45 (2.43)	0.009
CAT	12h	81.49±1.57	81.28±2.80	0.875
	48h	81.22±1.77	145.72±2.63	<0.001

Note: Shapiro–Wilk tests indicated that Treated 48 h subgroups of GSH-Px and T-SOD deviated from normal distribution. Accordingly, Mann–Whitney U test was adopted for pairwise comparisons between Control 48 h and Treated 48 h of these two indicators. All other comparisons followed normal distribution and were analyzed by Independent Samples t-test (n=6).
