# Supplementary material for: Physiological and Transcriptomic Responses to Cold Stress in Taiwan Loach (Paramisgurnus dabryanus ssp. Taiwan)
Source: Antioxidants (Basel). 2026 Aug 17;15(8):1022. doi: 10.3390/antiox15081022 (PMC13509272; doi:10.3390/antiox15081022)
Supplement: Supplementary file 1 [file antioxidants-15-01022-s001.zip › Table S3.pdf]

Table S3. KEGG pathway enrichment analysis of DEGs in Taiwan loach under cold stress.

Pathway	Padjust	Gene Number	Group
Fatty acid biosynthesis	0.00112	8	LT1
PPAR signaling pathway	0.00112	15	LT1
Glycerophospholipid metabolism	0.00112	18	LT1
Adipocytokine signaling pathway	0.003828	14	LT1
AMPK signaling pathway	0.010294	18	LT1
Fatty acid degradation	0.02359	9	LT1
Circadian rhythm	0.029856	8	LT1
Ribosome biogenesis in eukaryotes	6.70E-06	34	LT2
PPAR signaling pathway	0.001897	35	LT2
Spliceosome	0.002674	43	LT2
Tryptophan metabolism	0.002674	24	LT2
Arginine and proline metabolism	0.008141	24	LT2
Fatty acid biosynthesis	0.024117	12	LT2
Protein processing in endoplasmic reticulum	0.039484	48	LT2
Drug metabolism - other enzymes	0.039484	21	LT2
